# A case of rectal tumor in which the shape altered with regression in short period

**DOI:** 10.1186/1471-230X-13-146

**Published:** 2013-10-03

**Authors:** Fumihiko Nakamura, Taku Sakamoto, Takeshi Nakajima, Yutaka Saito, Hirokazu Taniguchi, Takahisa Matsuda

**Affiliations:** 1 Endoscopy Division, National Cancer Center Hospital, 5-1-1 Tsukiji, Chuo-ku, Tokyo 104-0045, Japan; 2 Digestive Disease Center, Kohseichuo General Hospital, 1-11-7 Mita, Meguro-ku, Tokyo 153-8581, Japan; 3 Pathology Division, National Cancer Center Hospital, 5-1-1 Tsukiji, Chuo-ku, Tokyo 104-0045, Japan

**Keywords:** Rectal tumor, Regression, Endoscopic submucosal resection with a ligation device (ESMR-L)

## Abstract

**Background:**

Histological regression of solid tumors in adults receiving no treatment is rare. Specifically, spontaneous partial and complete regression of colorectal cancers account for less than 2% of such cases and those without metastasis are exceedingly rare.

**Case presentation:**

A 60-year-old male underwent total colonoscopy following a positive fecal occult blood test at the referring hospital. A flat elevated lesion with central reddish depression, 10 mm in diameter, was detected in the lower rectum. Biopsy results from the referring hospital showed a well-differentiated adenocarcinoma and the patient was referred to our hospital for diagnosis and treatment. Preoperative colonoscopy was performed to determine the therapeutic strategy; however, we found only scar tissue and there were no endoscopic features to suggest malignancy. Biopsy from the scar revealed normal rectal mucosa and we performed diagnostic endoscopic submucosal resection with a ligation device (ESMR-L) one week later. The resected specimen showed a 1 mm well-differentiated adenocarcinoma with low-grade atypia and no lymphovascular invasion. The macroscopic type was 0-IIb, the depth of invasion was intramucosal, and the vertical and lateral margins were negative. There has been no evidence of recurrence for 18 months following treatment.

**Conclusion:**

We report a case of a rectal tumor showing regression over a short period without treatment. Spontaneous regression of malignant tumors is a rare and unexplained phenomenon. Further research and understanding of the mechanism holds the key for treatment and prevention of cancer in the future.

## Background

Histological regression of solid tumors in adults receiving no treatment is a rare phenomenon. However, the occurrence of this phenomenon does not necessarily mean that the cancer is cured; the regression may not be complete or permanent. In particular, spontaneous regression of primary colorectal cancer with no metastatic lesions is exceedingly rare. We report a case of an elderly man with spontaneous regression of a primary rectal tumor.

## Case presentation

**Figure 1:**
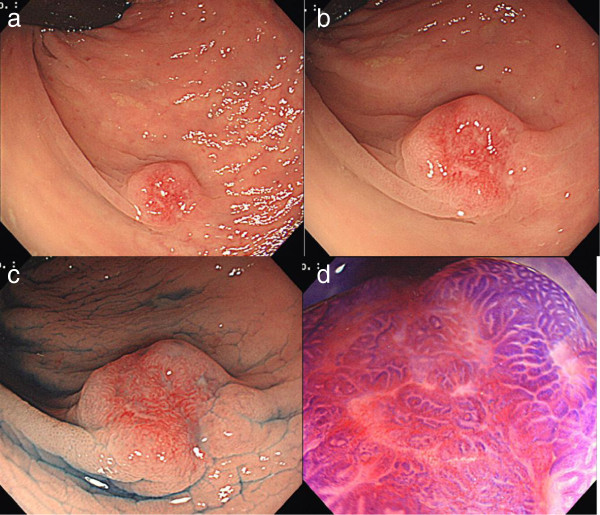
**Initial colonoscopy at the referring hospital. a**,**b** The lower rectal lesion was classified as IIa + IIc, 10 mm in size. The depressed area has a remarkable reddish surface with fold convergence. **c**. Magnifying chromoendoscopy with indigo carmine (0.4%) demonstrates the surface character and margins of the depressed area. **d**. Magnifying chromoendoscopy with crystal violet (0.05%) revealed Kudo’s type IIIL or VI mildly irregular pit pattern (non-invasive pattern in our clinical classification).

A 60-year-old male underwent total colonoscopy following a positive fecal occult blood test at a referring hospital. A flat elevated lesion with central depression, 10 mm in diameter, was detected in the lower rectum. The depressed area had a remarkable reddish surface with fold convergence (Figure 1a,b). Magnifying chromoendoscopy with indigo carmine demonstrated the surface character and the margins of the depressed area more clearly (Figure 1c). The depth of the lesion was predicted as submucosal (sm) invasion based on the conventional endoscopic findings because of the fold convergence and the reddish depressed area. However, staining with crystal violet revealed type IIIL and VI mildly irregular pit pattern according to Kudo’s classification [1,2] or non-invasive pattern in our clinical classification [3] (Figure 1d). Biopsy specimens revealed well-differentiated adenocarcinoma.

**Figure 2:**
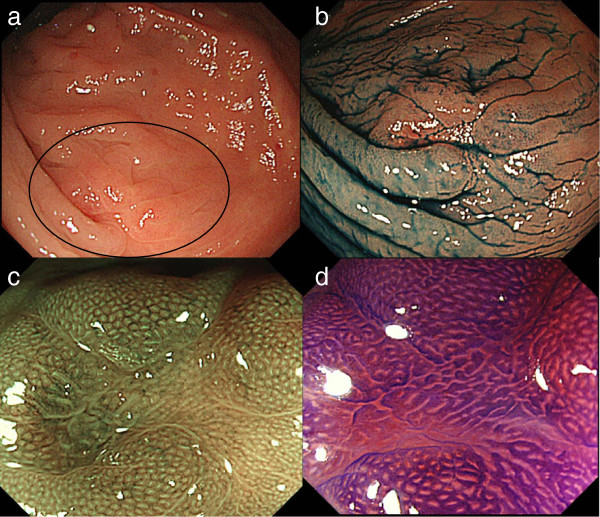
**First colonoscopy at our hospital. a**. Conventional (white light) image revealed an altered shape of the lesion. The lesion appeared smaller and more indistinct compared with that seen during the previous colonoscopy and it resembles a scar. **b**. Chromoendoscopic view with indigo carmine (0.4%) shows fold convergence but no apparent lesion. **c**. Narrow-band imaging with magnification revealed a faintly visible honeycomb pattern around the normal gland. This pattern was judged as Sano’s type I capillary pattern. **d**. Magnifying chromoendoscopy with crystal violet (0.05%) revealed a Kudo’s type IIIL or elongated type I pit pattern.

The patient was subsequently referred to our hospital for further management. Computed tomography for tumor staging showed no lymph node swelling or distant metastasis. Blood examination revealed increased serum carcinoembryonic antigen (CEA) levels (8.2 ng/ml) and normal carbohydrate antigen 19-9 (CA19-9) levels (< 1 U/ml) and a total colonoscopy was then performed in our hospital. A month after the previous colonoscopy, the examination showed different lesion morphology. Although the patient had received neither anticancer treatment nor any other medications, the lesion was smaller and more indistinct. However, the fold convergence persisted, suggesting a scar (Figure 2a). Chromoendoscopy with indigo carmine dye (0.4%) revealed a clear demarcation between the fold convergence and normal tissue; however, we were unable to detect the lesion clearly (Figure 2b). Narrow-band imaging (NBI) with magnification revealed a faintly visible honeycomb pattern around the normal gland. This finding was classified as type I according to Sano’s classification [4] (Figure 2c) and Kudo type IIIL or elongated type I pit pattern was observed with magnifying chromoendoscopy and crystal violet staining (0.05%) (Figure 2d). A biopsy taken from the center of the lesion revealed only normal rectal mucosa.

**Figure 3:**
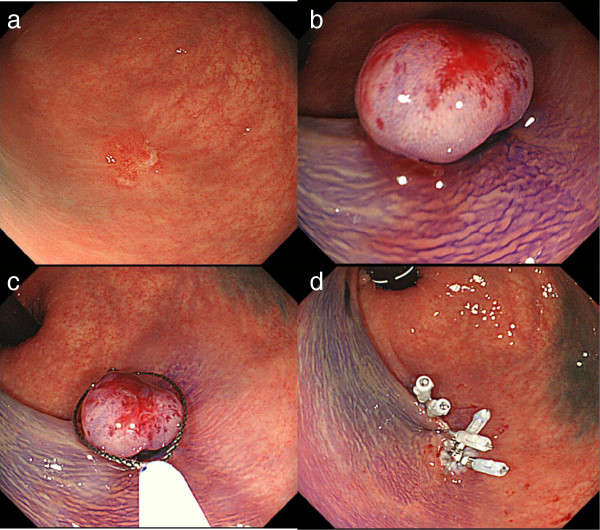
**The second colonoscopy was performed approximately one week after the first colonoscopy in our hospital. a**. The only observed lesion is a reddish mucosa with an ulcer probably caused by the previous biopsy. Demarcation of the lesion is unclear. **b**-**d** Endoscopic submucosal resection with a ligation device (ESMR-L) is performed with simultaneous *en-bloc* resection. Five clips are placed to obtain complete closure.

**Figure 4:**
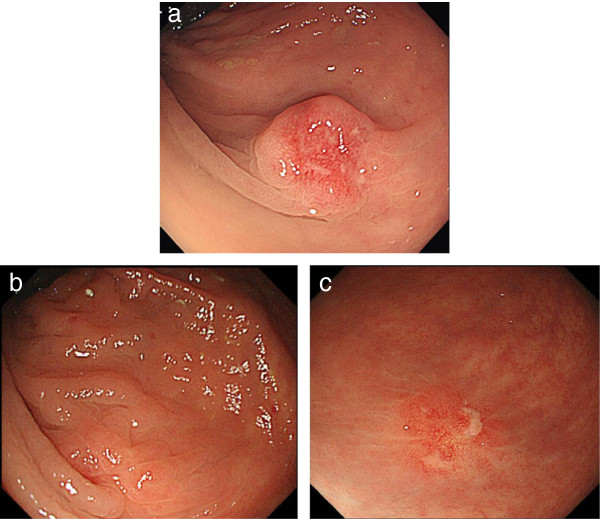
**Comparison of the colonoscopy findings.** On comparing these colonoscopy findings, the tumor seen during the colonoscopy performed at our hospital **(b**,**c)** appears smaller with indistinct features compared with the tumor seen during the colonoscopy performed at the referring hospital, one month earlier **(a)**.

Colonoscopy was repeated one week after the first examination at our hospital, and with this exam, there was reddish mucosa with erosion caused by the biopsy (Figure 3a). The endoscopic findings of the lesion revealed a remarkable reduction in size with the development of indistinct features during the short period of one month (Figure 4). Because it was difficult to diagnose the lesion by endoscopic findings and the biopsy specimen was normal, we performed diagnostic endoscopic submucosal resection with a ligation device (ESMR-L) (Figure 3b,c,d).

**Figure 5:**
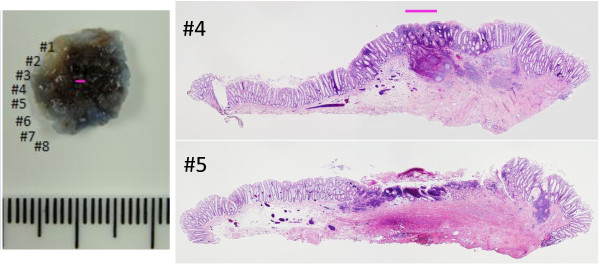
**Pathological findings (hematoxylin and eosin staining).** We divided the resected specimen into 8 sections. This figure shows panoramic views of sections No. 4 and No. 5. Intramucosal cancer is visible only in section No. 4 (pink line). In addition, remarkable fibrosis is visible over a broad range in sections No. 4 and No. 5.

**Figure 6:**
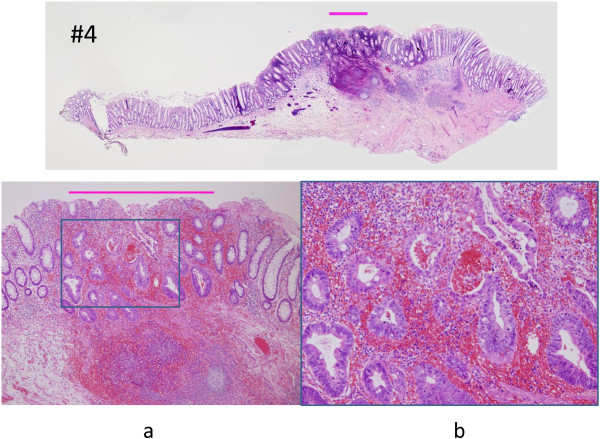
**Magnifying view of specimen No. 4. a**. The line of the muscularis mucosa was intricate. Cancer cells invaded the mucosal layer and muscularis mucosa, and remarkable congestion and inflammation are evident (hematoxylin and eosin stain, ×40). **b**. The intramucosal tumor was diagnosed as well-differentiated adenocarcinoma with low-grade atypia (hematoxylin and eosin staining, ×100).

Histopathological findings of the resected specimen showed a 1 mm well-differentiated adenocarcinoma with low-grade atypia and no lymphovascular invasion (the macroscopic type was 0-IIb). The depth of invasion was mucosal, and the vertical and lateral cut margins were negative. There was marked fibrosis, congestion, and inflammation around the intramucosal tumor (Figure 5, 6). Surveillance colonoscopy was performed and only scar tissue was observed at the site of resection. A biopsy taken from the scar revealed normal rectal mucosa. The patient is currently in remission with no recurrence detected after 18 months of follow up.

## Discussion

Spontaneous regression is characterized by partial or complete degeneration of a tumor without any treatment [5,6]. However, the occurrence of regression does not necessarily mean that the tumor is cured and the regression may not be complete or permanent. Spontaneous regression of a malignant tumor is rare, occurring in one in 60,000-100,000 cases, and approximately 3 cases are reported every year from around the world [7,8]. Seventy percent of regressed cases occur in renal cancers, neuroblastoma, malignant melanoma, bladder cancer, or soft tissue sarcomas. Regression of colorectal cancer is exceedingly rare [9], and represents less than 2% of all cases of spontaneous regression [10].

Spontaneous tumor regression could potentially be initiated by immune factors such as tumor necrosis factor-α, tumor growth factor-β, α-interferon, and natural killer cells; induction of differentiation; hormonal mediation; elimination of a carcinogen; tumor necrosis or apoptosis; angiogenesis inhibition; and external factors such as biopsy, infection, trauma, and surgery [11]. Generally, ischemia-or necrosis-activated immune responses initiate tumor regression. Ischemic change in the tumor is caused by vessel obstruction or hypoxia, which depends on the growth rate of the tumor, and shock caused by hemorrhage or dehydration.

Initially, this patient presented at the referring hospital with a tumor measuring 10 mm in size. However, colonoscopy at our hospital, which was performed approximately 4 weeks after the previous colonoscopy, showed that the tumor had regressed with only a scar remaining. Finally, the resected specimen revealed only a 1 mm small mucosal cancer.

Biopsy is thought to be a major contributing factor for tumor regression in this case. The tumor had a remarkably altered shape in a short period. Also, microscopy showed not only congestion and immune cells but also widespread, marked fibrosis around the tumor. Therefore, it was difficult to presume that biopsy was the only causative factor for the altered shape of the lesion and the indications were that this rectal lesion was formed not only by biopsy but also by other factors. Factors such as immunity activated by physical stimulation, inflammation, and ischemic change based on tumor growth, as well as biopsy, could have led to regression in the present case. Ayman S *et al*. [12] reviewed 21 reported cases of spontaneous regression of colorectal cancer from 1900-2005 and found that regression of primary lesions or local recurrences was confirmed in only 14 (66.7%) cases (other cases accounted for regression of liver metastasis or peritoneal carcinomatosis). Six of these 14 cases (42.9%) were located in the rectum, as in the present case, whereas the others occurred in the ascending colon (14.3%), transverse colon (14.3%), descending colon (7.1%), and sigmoid colon (21.4%). These findings indicate that the rectum, affected by physical stimulation such as residue or stool, is a preferred site for regression of colorectal cancer.

This case also validated the utility of ESMR-L for full staging and diagnosis of difficult lesions. Endoscopic resections such as polypectomy or conventional endoscopic mucosal resection (EMR) are simple and less invasive procedures. However, complete resection of a scar-like lesion, as in the present case, is difficult using these techniques irrespective of tumor size. Mashimo *et al*. reported that ESMR-L is superior to conventional EMR or polypectomy for complete resection of rectal carcinoid tumors. Because the wall of the lower rectum is thick and supported by surrounding connective tissue, ESMR-L can be performed with full suction to achieve a deeper vertical margin without perforation, thus providing a higher complete resection rate even when the lesions comprise scar tissue or are small in size, as in the present case. The complete resection rate for tumors of the lower rectum is reportedly 98.3% [13]. We made an accurate diagnosis using ESMR-L for a scar-like lesion with remarkable fibrosis in the present case, as in other reported cases.

It is important to note that regression in the present case occurred despite the absence of anticancer treatment or any other medication until the patient underwent endoscopic treatment. Further identification and characterization of similar cases could result in defining a patient population that can be followed by observation, aiding in elucidating this phenomenon further.

The rarity of spontaneous regression in cases of colorectal cancer can be attributed to immediate administration of treatment following diagnosis, as technological advances now allow removal of early-stage colorectal tumors at the time of endoscopic examination.

## Conclusion

We report a case of a rectal tumor that exhibited alteration of shape with regression over a short period of one month. Spontaneous regression of a malignant tumor is a rare and interesting phenomenon. A better understanding of the underlying mechanism will be a key factor for treatment and prevention of cancer in the future. The present case may provide valuable information that can aid in the development of novel therapeutic strategies for the treatment and prevention of cancer.

## Consent

Written informed consent was obtained from the patient for publication of this case report and the accompanying images. A copy of the written consent is available for review by the editor-in-chief of this journal.

## Abbreviations

ESMR-L: Endoscopic submucosal resection with a ligation device; sm: Submucosal; NBI: Narrow-band imaging; CEA: Carcinoembryonic antigen; CA 19-9: Carbohydrate antigen 19-9; EMR: Endoscopic mucosal resection.

## Competing interests

The authors declare that they have no competing interests.

## Authors’ contributions

FN designed and drafted the manuscript; Dr. TS was responsible for the revision of the manuscript and the supervision of the endoscopic diagnosis; Dr. TN and Dr. YS were responsible for the endoscopic diagnosis; Dr. HT made the pathological diagnosis; and Dr. TM revised the manuscript and was responsible for the endoscopic diagnosis and treatment. All authors approved the final manuscript.

## Pre-publication history

The pre-publication history for this paper can be accessed here:

http://www.biomedcentral.com/1471-230X/13/146/prepub
